# Biocompatibility and Osteogenic Capacity of Mg-Zn-Ca Bulk Metallic Glass for Rabbit Tendon-Bone Interference Fixation

**DOI:** 10.3390/ijms20092191

**Published:** 2019-05-03

**Authors:** Chin-Chean Wong, Pei-Chun Wong, Pei-Hua Tsai, Jason Shian-Ching Jang, Cheng-Kung Cheng, Hsiang-Ho Chen, Chih-Hwa Chen

**Affiliations:** 1 Department of Orthopedics, Shuang Ho Hospital, Taipei Medical University, Taipei 23561, Taiwan; b8701153@tmu.edu.tw; 2 Department of Orthopedics, School of Medicine, College of Medicine, Taipei Medical University, Taipei 11031, Taiwan; 3 Department of Biomedical Engineering, National Yang-Ming University, Taipei 11221, Taiwan; s0925135546@gmail.com (P.-C.W.); ckcheng2009@gmail.com (C.-K.C.); 4 School of Biomedical Engineering, College of Biomedical Engineering, Taipei Medical University, Taipei 11031, Taiwan; 5 Institute of Materials Science and Engineering; Department of Mechanical Engineering, National Central University, Taoyuan 32001, Taiwan; peggyphtsai@gmail.com; 6 Department of Orthopedics, Bone and Joint Research Center, Taipei Medical University Hospital, School of Medicine, College of Medicine, Taipei Medical University, Taipei 11031, Taiwan

**Keywords:** MgZnCa bulk metallic glass, biocompatible, biodegradable, osteogenic

## Abstract

Mg-based alloys have great potential for development into fixation implants because of their highly biocompatible and biodegradable metallic properties. In this study, we sought to determine the biocompatibility of Mg_60_Zn_35_Ca_5_ bulk metallic glass composite (BMGC) with fabricated implants in a rabbit tendon–bone interference fixation model. We investigated the cellular cytotoxicity of Mg_60_Zn_35_Ca_5_ BMGC toward rabbit osteoblasts and compared it with conventional titanium alloy (Ti6Al4V) and polylactic acid (PLA). The results show that Mg_60_Zn_35_Ca_5_ BMGC may be classed as slightly toxic on the basis of the standard ISO 10993-5. We further characterized the osteogenic effect of the Mg_60_Zn_35_Ca_5_ BMGC extraction medium on rabbit osteoblasts by quantifying extracellular calcium and mineral deposition, as well as cellular alkaline phosphatase activity. The results of these tests were found to be promising. The chemotactic effect of the Mg_60_Zn_35_Ca_5_ BMGC extraction medium on rabbit osteoblasts was demonstrated through a transwell migration assay. For the in vivo section of this study, a rabbit tendon–bone interference fixation model was established to determine the biocompatibility and osteogenic potential of Mg_60_Zn_35_Ca_5_ BMGC in a created bony tunnel for a period of up to 24 weeks. The results show that Mg_60_Zn_35_Ca_5_ BMGC induced considerable new bone formation at the implant site in comparison with conventional titanium alloy after 24 weeks of implantation. In conclusion, this study revealed that Mg_60_Zn_35_Ca_5_ BMGC demonstrated adequate biocompatibility and exhibited significant osteogenic potential both in vitro and in vivo. These advantages may be clinically beneficial to the development of Mg_60_Zn_35_Ca_5_ BMGC implants for future applications.

## 1. Introduction

Magnesium (Mg) alloys have emerged as ideal candidates for the surgical fixation of implants because of their highly biocompatible and biodegradable metallic properties. Compared with other conventional materials used for surgical fixation, such as stainless steel or titanium alloy (Ti6Al4V), Mg-based alloys have Young’s moduli similar to bone, thus lowering the risk of the load-shielding phenomenon after in vivo implantation [1]. Biodegradability is another major advantage of Mg-based alloys, as there is no need for a second operation to remove the implant after bony union is achieved. Despite its biodegradability, Mg-based alloys have a notably rapid degradation rate, as reported by previous studies [2,3]. Accordingly, magnesium aqueous hydroxide and hydrogen gas are produced during the degradation process. Hydrogen gas production, a change in pH, and the degradation products of magnesium (Mg(OH)_2_) all accompany this process; they have adverse effects on the viability of cells and may induce tissue inflammation. In contrast, Mg_60_Zn_35_Ca_5_ bulk metallic glass composite (BMGC) has been reported to exhibit a degradation rate much lower than those of traditional Mg-based crystalline alloys because of its single-phase structure [4]. The in vitro degradation behavior of Mg_60_Zn_35_Ca_5_ BMGC was evaluated well in our previous work [4]. The dispersion of titanium particles within the composite markedly improve the compressive strength and brittleness of Mg_60_Zn_35_Ca_5_ BMGC relative to those of conventional biodegradable implants made of synthetic polymers, rendering it a suitable candidate for developing load-bearing bone fixation devices [5].

Furthermore, studies have demonstrated that Mg^2+^ ions released during Mg-based BMGC degradation could display significant biological effects such as enhancing new regional bone formation, as well as promoting osteoblast proliferation activity and migratory capacity, in addition to the improved mechanical properties of Mg alloy [6]. A recent study conducted by Yoshizawa et al. further confirmed the stimulatory effect of magnesium ions on the osteogenic activity in bone marrow stromal cells both in vitro and in vivo [7,8,9]. Moreover, Mg^2+^ ions can stimulate osteoblast proliferation and migration through melastatin-like transient receptor potential 7 (TRPM7) channels by Mg^2+^ influx pathway mediated by platelet-derived growth factor (PDGF) [8]. These results indicate the potential of these materials for use in future craniofacial and orthopedic applications. From these studies, it was believed that Mg_60_Zn_35_Ca BMGC can have similar biological effects on bone tissues by releasing magnesium ions after in vivo implantation.

In this study, we analyzed the biocompatibility and osteogenic effects of Mg_60_Zn_35_Ca BMGC as a function of osteoblast survival rate and extracellular osteogenic mineral deposition. To examine the in vivo behavior of this material, rods fabricated with Mg_60_Zn_35_Ca BMGC were implanted into a pre-drilled bone tunnel in rabbits. We hypothesized that this rabbit tendon–bone fixation model would allow us to closely study the in vivo biocompatibility and osteogenic potential of the composite rods. The results of these tests may provide important evidence of the potential use of Mg_60_Zn_35_Ca_5_ BMGC for future surgical implant developments and applications.

## 2. Result

### 2.1. Cell Viability

**Figure 1:**
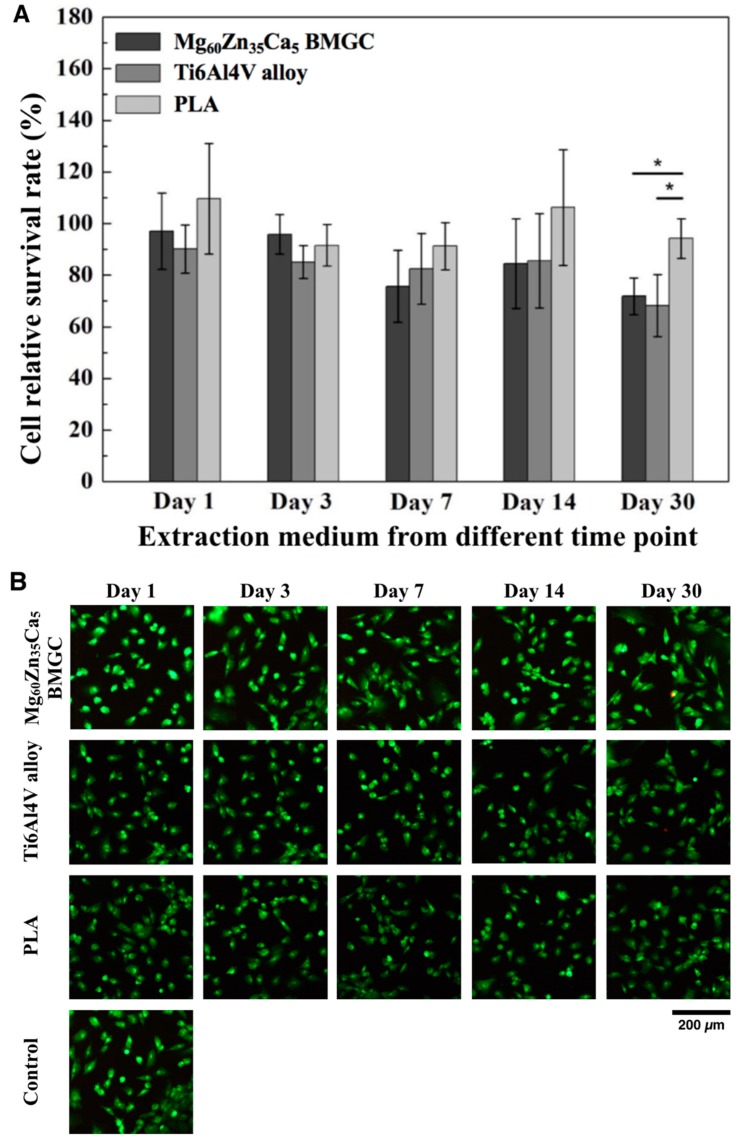
Cell viability of primary rabbit osteoblasts cultured in either Mg_60_Zn_35_Ca_5_, Ti6Al4V alloy or PLA-derived extraction medium for different periods. (**A**) MTT assay. (**B**) Live/dead assay. (* *p* < 0.05).

The cell viability of the rabbit primary osteoblasts (normalized against the control group) cultured in media with Mg_60_Zn_35_Ca_5_ BMGC, Ti6Al4V alloy, or PLA for different lengths of time are shown in Figure 1. No significant difference of cell viability was observed among three groups cultured with extraction medium obtained from Day 1 to Day 14. However, cells cultured with the PLA extraction medium obtained from Day 30 had significant higher cell viability compared with the other groups (*p* < 0.05). Nonetheless, the cell viability of all groups can be classified as the first level cytotoxicity according to ISO-10993-5 [10]. In Figure 1B, the live/dead immunofluorescence results are compatible with the MTT assay showing a decline of cell viability in osteoblasts cultured with extraction medium from Day 30.

### 2.2. ALP Activity

**Figure 2:**
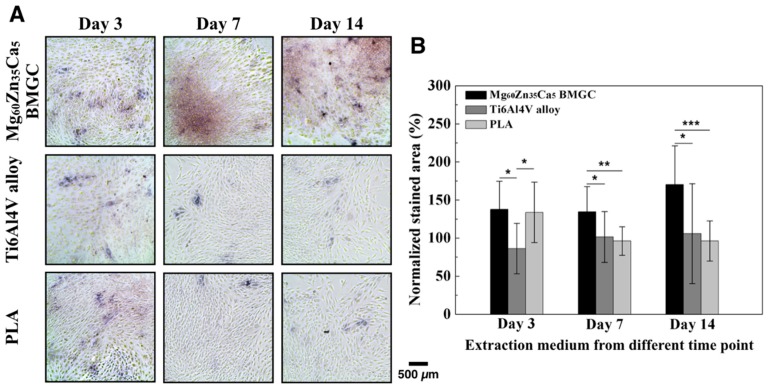
Alkaline phosphatase (ALP) activity of primary rabbit osteoblasts under treatment with different extraction medium. (**A**) Image of ALP staining. (**B**) Quantitation of ALP of primary rabbit osteoblasts cultured in different extraction medium for different time periods. (Data normalized with control group: without extraction medium; * *p* < 0.05, ** *p* < 0.01, *** *p* < 0.001.)

ALP is a bone matrix protein that can help further form and synthesize the bone matrix, as well as aid in the formation of collagen type 1 alpha 1 (Col1α1) and osteocalcin (OC) [11]. ALP activity increases with increased osteoblastic activity; thus, the function of osteoblasts can be evaluated via ALP activity testing. Figure 2A shows the images that were obtained after staining; the area stained purple indicates ALP. Osteoblasts cultured with Mg_60_Zn_35_Ca_5_ BMGC extracted medium showed a much higher ALP activity than those cultured with Ti6Al4V alloy and PLA extracted media. The percentage of stained area was quantified by Image J software and normalized using the untreated group, as shown in Figure 2B. The highest ALP activity was that of Mg_60_Zn_35_Ca_5_ BMGC with a high concentration of extracted medium (day 14), and it was dose-dependent.

### 2.3. Extracellular Matrix Calcium Deposition

**Figure 3:**
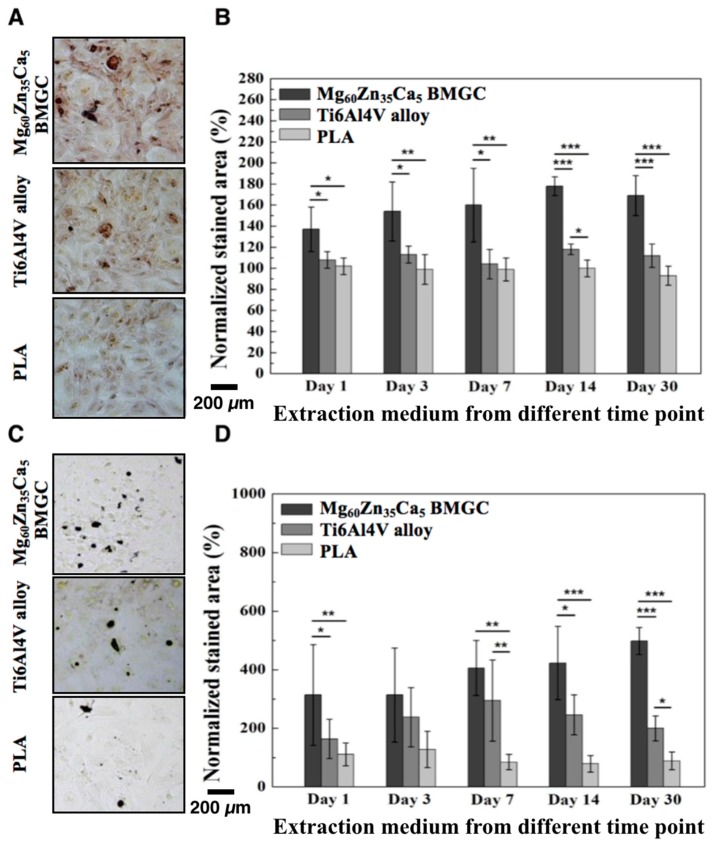
Extracellular calcium and mineral deposition by rabbit osteoblasts under treatment with different extraction medium. (**A**) Image of alizarin red S staining. (**B**) Quantitation of calcification of the extracellular matrix of primary rabbit osteoblasts cultured in different extraction medium for different time periods that stained by alizarin red S staining. (**C**) Image of von Kossa staining. (**D**) Quantitation of mineralization of primary rabbit osteoblasts cultured in different extraction medium for different time periods that stained by von Kossa staining. (Data normalized with control group: without extraction medium; * *p* < 0.05, ** *p* < 0.01, *** *p* < 0.001.)

The extracellular matrix (ECM) calcification of rabbit primary osteoblast cells were evaluated through ARS staining. Images of the stained cell layers are shown in Figure 3A; the area stained dark red indicating intense calcification deposition can be observed in the images. Osteoblasts cultured with the Mg_60_Zn_35_Ca_5_ BMGC extracted medium showed calcification deposition significantly (*p* < 0.001) more than that of the PLA extracted medium. The ratio of the stained area to the entire area of the culture well was quantified using Image J software for each extracted medium, as shown in Figure 3B. The stained area of each group was normalized with the control group (without the simulation of any extracted medium). The percentage of ECM calcification in the osteoblasts simulated using different materials and different concentrations of the extracted medium was 137 ± 21% for Mg_60_Zn_35_Ca_5_ BMGC, 108 ± 8% for Ti6Al4V alloy, and 102 ± 8% for PLA at low concentrations of the extracted media (day 1). Thereafter, the stained areas of Mg_60_Zn_35_Ca_5_ BMGC, Ti6Al4V alloy, and PLA were 169 ± 19%, 112 ± 11%, and 93 ± 9%, respectively, at high concentrations of the extracted medium (day 30). Overall, the ECM calcification significantly increased (*p* < 0.05) in groups simulated with Mg_60_Zn_35_Ca_5_ BMGC, for both low and high concentrations of the extracted medium.

The calcium deposition of the rabbit primary osteoblasts was visualized by the dark brown and black colors obtained from von Kossa staining. As shown in Figure 3C, a smaller stained area appeared in cells that were cultured with the PLA extracted medium, while the osteoblast cells cultured with the Mg_60_Zn_35_Ca_5_ BMGC extracted medium demonstrated a significantly larger (*p* < 0.001) stained area. The percentage of the stained area to the entire area of the culture well was quantified by Image J software for each extracted medium, as shown in Figure 3D. The stained area of each group was normalized with the control group (simulated without any extracted medium). The percentages of the calcium deposition of the osteoblasts that were simulated with different concentrations of the extracted media were 314 ± 236% for Mg_60_Zn_35_Ca_5_ BMGC, 164 ± 67% for Ti6Al4V alloy, and 111 ± 39% for PLA at a low concentration of the extracted media (day 1). Thereafter, the stained areas of Mg_60_Zn_35_Ca_5_ BMGC, Ti6Al4V alloy, and PLA medium extracts were 498 ± 46%, 200 ± 42%, and 89 ± 30%, respectively, at high concentrations (day 30). In general, the Mg_60_Zn_35_Ca_5_ BMGC led to calcium deposition of osteoblasts significantly greater than that in the PLA group (*p* < 0.001).

### 2.4. Migration Capacity

**Figure 4:**
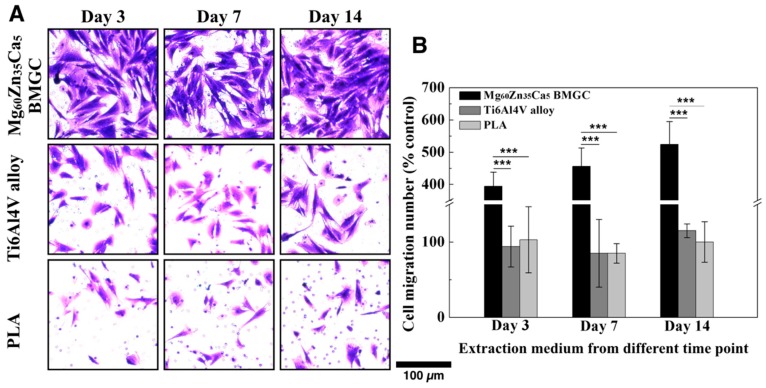
Migration capacity of primary rabbit osteoblasts under treatment with different extraction medium. (**A**) Image of migrated cells stained with crystal violet. (**B**) Quantitation of migrated cell number of primary rabbit osteoblasts cultured in different extraction medium for different time periods. (Data normalized with control group: without extraction medium; *** *p* < 0.001.)

In terms of shortening the period of bone healing, the recruitment of cells from the surrounding tissue by the implanted materials is a positive and important issue. For the bone healing process, the attraction of cells that surround the damaged or defective tissue does not only enhance the function of these cells, but also increases the number of cells and quickens the healing progress. Therefore, it is imperative to test the migration capacity of Mg_60_Zn_35_Ca_5_ BMGC, Ti6Al4V alloy, and PLA. After 12 h of incubation followed by medium extraction, the images of the migrated osteoblasts were stained and captured by an optical microscope, as shown in Figure 4A. As clearly seen in Figure 4A, the number of migrated cells in the Mg_60_Zn_35_Ca_5_ BMGC group was higher than those in the other groups. The imaging results were quantified by Image J software and normalized with the control (untreated) group (Figure 4B). Whether in low (day 3), medium (day 7), or high (day 14) concentrations of the extraction medium, the Mg_60_Zn_35_Ca_5_ BMGC group demonstrated the highest cell migration capacity at values of 394 ± 44%, 456 ± 57%, and 524 ± 71%, respectively.

### 2.5. Cell Morphology Observation

**Figure 5:**
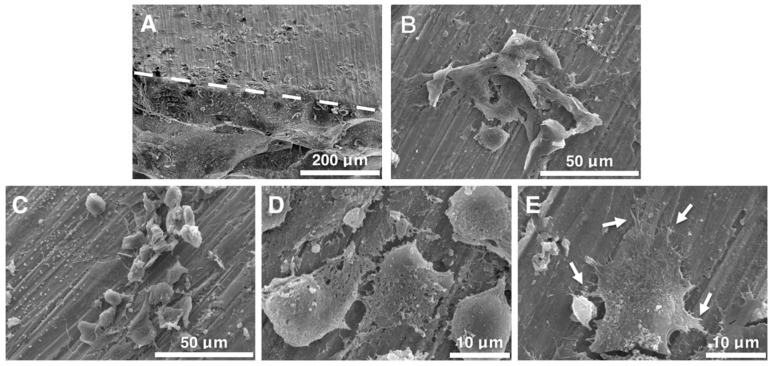
SEM images of MG63 cell line cultured for 12 h on Mg_60_Zn_35_Ca_5_ BMGC. (**A**) MG63 cells adhesion on two vertical solid surfaces. (**B**–**D**) MG63 cells were stacked on the surface of Mg_60_Zn_35_Ca_5_ BMGC with good adhesion and spreading morphology. Cells were not only attachment between pseudopodia and the surface of Mg_60_Zn_35_Ca_5_ BMGC but also morphology of cell-cell interaction can be observed clearly. (**E**) The cytoskeleton of MG63 cells was well expanded and presented a pronounced spreading and formation of pseudopodia on surface of Mg_60_Zn_35_Ca_5_ BMGC (indicated by arrow).

Figure 5 shows the cell morphology images which were captured by SEM. In Figure 5A–C, stacked MG63 cells show good adhesion and exhibiting well spindle shape on Mg_60_Zn_35_Ca_5_ BMGC surface, cells were not only displayed two-dimension structure but also performing three-dimension structure. Moreover, the cell–cell interaction can also be observed clearly. Figure 5D presented that the excellent expanded cytoskeleton of MG63 cells, the evidence of attached tightly was the pseudopodia formation and spreading pronouncedly on the surface of Mg_60_Zn_35_Ca_5_ BMGC (indicated by arrow).

### 2.6. Radiological Evaluation

**Figure 6:**
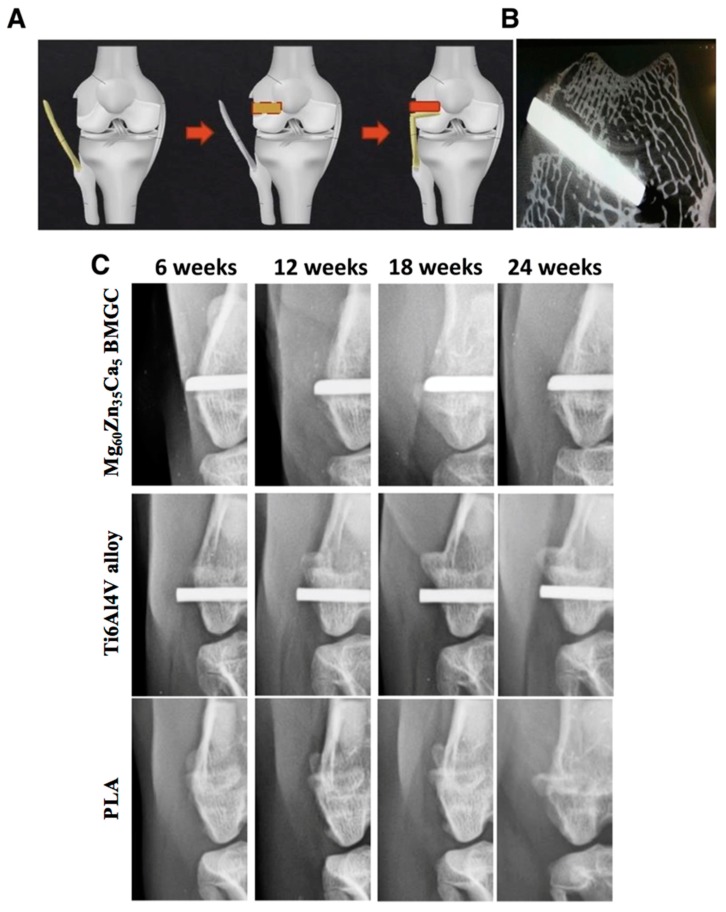
(**A**) Figure illustration of rabbit femur tendon-bone interference fixation model. First, lateral collateral ligament was detached from its femoral attachment. A 2.4 mm drill was used to create a bone tunnel which was 10 mm in length. Then, the tendon was fixed into the bone tunnel and secured with a rod acting as an interference implant. (**B**) Axial micro-CT image showing an implanted rod resided inside the bone tunnel. (**C**) Radiographic images of the rabbit’s femur implanted with either Mg_60_Zn_35_Ca_5_ BMGC, Ti6Al4V alloy, or PLA materials at different time points.

Figure 6A shows a rabbit femur tendon–bone interference fixation model. Figure 6B shows a representative axial micro-CT image of an implanted rod residing inside the created bone tunnel. Figure 6C shows the representative radiographs of the distal femur with the implantation of either Mg_60_Zn_35_Ca_5_ BMGC, Ti6Al4V alloy, or PLA at 6, 12, 18, and 24 weeks postoperatively. The radiographic results show that all metallic implants were fixed well inside the bony tunnel throughout the follow-up period. Specifically, no radiolucent clear zones suggesting gaseous formation were noted at the implant sites. Nonetheless, the position of the PLA implants could not be visualized via conventional radiographs because of the inherent non-radiopaque feature of this material.

### 2.7. Micro-CT Image (Bone Mineral Density and 3D Image Reconstruction)

**Figure 7:**
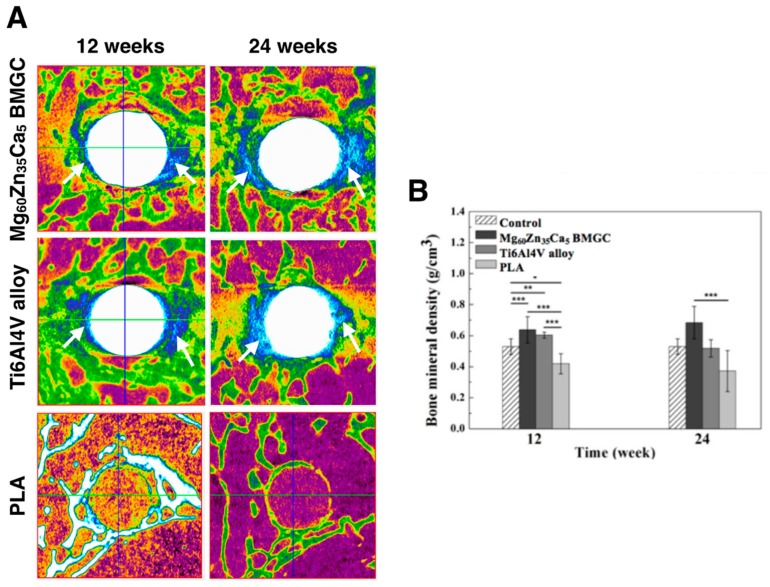
(**A**) Micro-CT image of the rabbit’s femur implanted with either Mg_60_Zn_35_Ca_5_ BMGC, Ti6Al4V alloy, or PLA materials at 12 and 24 weeks postoperatively. (**B**) Intergroup comparison of bone mineral density surrounding the implanted site at 12 and 24 weeks, analyzed with CTan analyzer software (* *p* < 0.05, ** *p* < 0.01, *** *p* < 0.001).

The micro-CT images in Figure 7A show the Mg_60_Zn_35_Ca_5_ BMGC, Ti6Al4V alloy, and PLA implantations at 12 and 24 weeks post-operation. None of the micro-CT images revealed gaseous formation within the bony tunnel at 12 and 24 weeks. In the Mg_60_Zn_35_Ca_5_ BMGC and Ti6Al4V alloy groups, significant new bone formation around the rod was noted at 12 and 24 weeks after implantation. Very limited new bone formation could be detected in PLA group, regardless of the implantation period.

For new bone formation quantitation, the bone mineral density (BMD) around the implant sites were analyzed by CTan analyzer software (Bruker), as shown in Figure 6B,C. The intergroup comparison of the BMD surrounding the implant sites showed that the Mg_60_Zn_35_Ca_5_ BMGC and Ti6Al4V alloy groups achieved significantly levels of BMD (*p* < 0.001) higher than those of the control and PLA groups, at both 12 and 24 weeks of implantation (Figure 7B). Interestingly, we found that the BMD of the Ti6Al4V alloy group for the metallic implant groups decreased from week 12 to week 24, but this phenomenon was not observable in the Mg_60_Zn_35_Ca_5_ BMGC group. This implies the existence of more sustainable osteo-promoting effects following the release of Mg ions in situ.

### 2.8. Histology Observation

**Figure 8:**
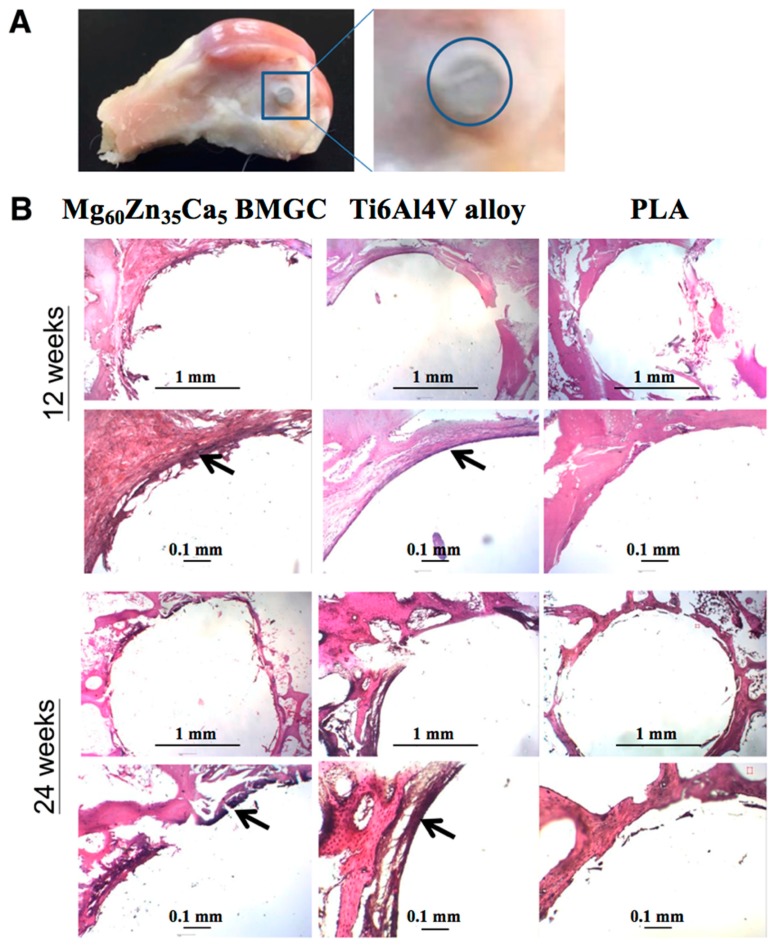
(**A**) Gross image of harvested rabbit femur indicating the surgical implanted site. The fixation rod was first removed before sent for histologic section. (**B**) Histological images of the implanted site at 12 weeks and 24 weeks. Black arrows indicate new bone formation. (Hematoxylin and eosin staining, upper panel 10×, lower panel 20×.)

Figure 8A shows the representative gross sagittal image of a harvested rabbit femur exposing the surgical implant site. All the implanted rods were found to be well-fixed inside the bone tunnel with no evidence of loosening. Before they were sent for histological sectioning, all rods were removed manually. Microscopically, specific attention was paid to determine new bone formation at the interface between the surrounding bone and the implanted materials by H&E staining. The results show remarkable new bone formation surrounding the Mg_60_Zn_35_Ca_5_ BMGC and Ti6Al4V alloy at 12 and 24 weeks after implantation (Figure 8B). At a higher magnification, many osteoblasts were found surrounding the Mg_60_Zn_35_Ca_5_ BMGC and Ti6Al4V alloy rods. In contrast, the interface between the bone tissue and the rods made from PLA was smooth, regardless of the implantation period, indicating very limited new bone formation. No inflammatory cells such as leukocytes or macrophages were localized at the implant sites, signifying good biocompatibility for all three materials.

### 2.9. Hematology Analysis

**Figure 9:**
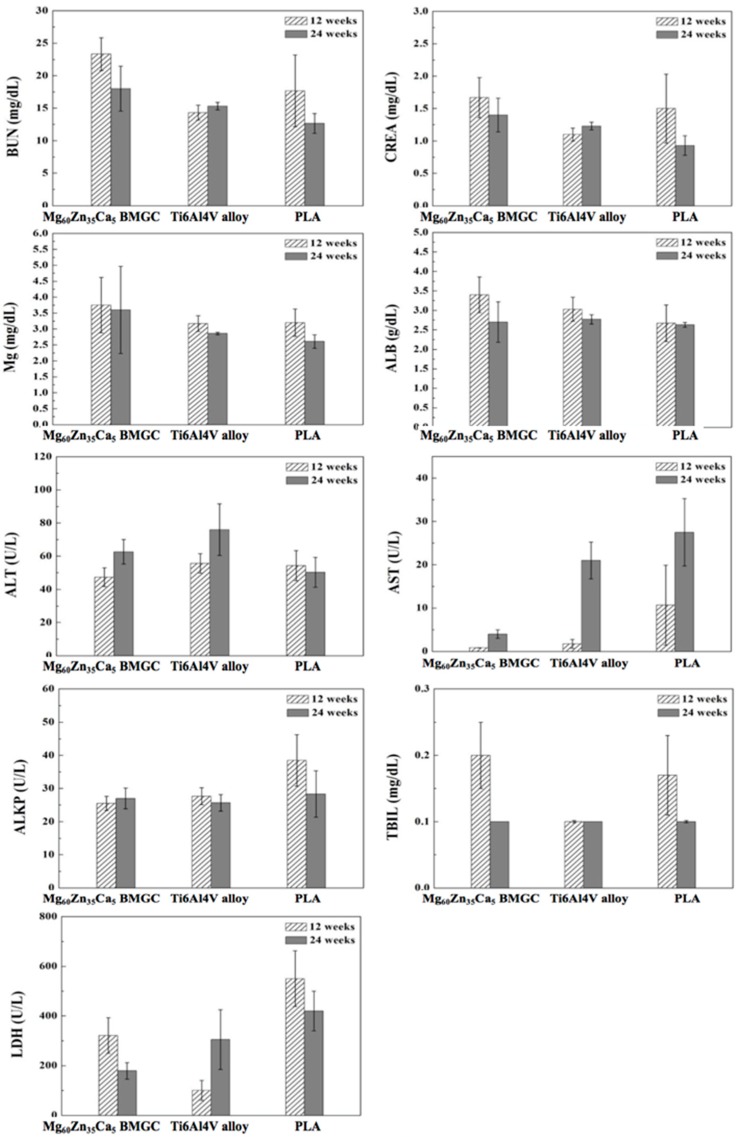
Blood chemical analysis of Mg_60_Zn_35_Ca_5_ BMGC, Ti6Al4V alloy, and PLA at 12 and 24 weeks postoperative (BUN, CREA, Mg, ALB, ALT, AST, ALKP, TBIL, and LDH).

At 12 and 24 weeks after the implantation of Mg_60_Zn_35_Ca_5_ BMGC, Ti6Al4V alloy, and PLA, no rabbit displayed significant local inflammation. As shown in Figure 9, at each time point, the measured levels of BUN, CREA, serum Mg, ALB, ALT, AST, ALKP, TBIL, and LDH among animals in each group were compared and showed no significant difference.

## 3. Discussion

Mg_60_Zn_35_Ca_5_ BMGC has an amorphous structure like other metallic glass materials, in which the atoms either have short-range ordering or are completely disordered. Moreover, there are no crystalline defects such as dislocations or gain boundaries. As a result, Mg_60_Zn_35_Ca_5_ BMGC has increased tensile strength, elastic energy, and hardness, as well as good corrosion and wear resistances. In this study, Mg_60_Zn_35_Ca_5_ BMGC was tested to evaluate its biocompatibility as compared with commercial Ti6Al4V alloy and PLA. We used a higher ion concentration by immersing the tested materials for a longer period in the culture medium (up to 30 days) for primary rabbit osteoblast cultivation. The results show that low concentrations of all samples did not cause cellular cytotoxicity. However, when the ion concentration increased (at an immersion time of 30 days), minor cytotoxicity to osteoblasts was apparent in the Mg_60_Zn_35_Ca_5_ BMGC and Ti6Al4V alloy groups. Mg_60_Zn_35_Ca_5_ BMGC can offer a suitable surface for cells adhesion, proliferation and further stacked to formed three-dimension morphology. We thus speculated that the lower degradation rate achieved with Mg-based BMGCs is accompanied by the release of significantly less metallic ions when implanted in vivo.

During the degradation of Mg_60_Zn_35_Ca_5_ BMGC and PLA, some ions are released and absorbed by the body. These ions are circulated through the blood and body fluids, and are stored in muscle, bone, serum, and in different types of cells. Excessive amounts of unabsorbed magnesium ions in the body are excreted through urine. The liver and kidneys are responsible for excretion and detoxification; thus, the serum magnesium concentration, along with the BUN, CREA, ALB, ALT, AST, ALKP, TBIL, and LDH indices, were checked. The authors wanted to ensure that the released ions would not reach a toxic level that may affect an animal’s renal and liver functions. According to the results (Figure 6), there was no significant difference in the serum’s biochemical parameters throughout the follow-up period (up to 24 weeks) among the three groups of animals. All rabbits demonstrated good health before sacrifice with no evidence of local (surgical site) or systemic inflammation. The results show that all of the implanted materials are biocompatible in vivo.

Most of the conventionally used fixation implants provide good mechanical strength and durability, but none has osteogenic and osteoconductive potentials in the host tissue. It was reported that magnesium ions could stimulate and enhance the osteogenic activity of bone marrow stromal cells [7]. The mineralization of the ECM, along with collagen type X mRNA and protein were found to be enhanced after treatment with 10 mM MgSO_4_ solution. To further characterize the osteogenic effects of Mg_60_Zn_35_Ca_5_ BMGC on rabbit osteoblasts, we prepared an extraction medium by immersing different materials in a standard culture medium for different durations. It is well known that increasing amounts of ions are be released after prolonged immersion. ARS and von Kossa histochemical staining were then used to investigate the amount of extracellular calcium deposition after induction with different concentrations of the extraction medium. We used 5-bromo-4-chloro-3-indolyl phosphate (BCIP)/nitro-blue tetrazolium (NBT) substrate staining to visualize the ALP activity of the osteoblasts. The results show that Mg_60_Zn_35_Ca_5_ BMGC achieved significantly higher levels of calcium deposition and ALP activity compared with the Ti6Al4V alloy and PLA groups (Figure 2B and Figure 3B,D), as the concentration of the extraction medium increased. In addition, calcium deposition showed a concentration-dependent phenomenon in the Mg_60_Zn_35_Ca_5_ BMGC group, a result that agrees with the findings of our previous studies [9]. In contrast, PLA exhibited a very limited osteo-promoting effect in terms of extracellular calcium and mineral depositions. For tendon–bone interference fixation to occur, the dense bone tissue must form entirely around the fixation implant. This is achieved by not only increasing cell proliferation, but also by recruiting osteoblasts from the surrounding tissue. Calcium deposition may be the result of the initial behavior and response of osteoblasts, and it must be stepwise, e.g., via migration followed by the attachment and proliferation of cells; finally, the osteoblasts can promote the deposition of calcium [12]. The results of this study show that the migration capacity of osteoblasts for the Mg_60_Zn_35_Ca_5_ BMGC group was significantly higher than that of the other groups (Figure 4A,B).

Several studies have shown that magnesium alloy orthopedic implants can increase bone mass, enhance the mineral apposition rate, and increase BMD [3,13,14,15,16]. Micro-CT scanning and reconstruction processes could effectively analyze BMD changes between time intervals. The BMD value after implantation was compared with the BMD value in normal rabbits (the reference). At 12 weeks post-operation, all implants made from the different materials had trabecular bone growth attached to the periphery of the implant (Figure 7A). According to the quantitated data (Figure 7B,C), the BMD values of Mg_60_Zn_35_Ca_5_ BMGC and Ti6Al4V alloy were significantly higher (*p* < 0.001) than that of PLA at 12 weeks post-operation. At 24 weeks post-operation, there was no statistical difference in BMD between Mg_60_Zn_35_Ca_5_ BMGC and Ti6Al4V alloy.

In addition to BMD, histological observation is another way to observe osteointegration. Bone remodeling and growth was observed around all rods at 12 weeks after implantation. At 12 weeks, the degradation materials included in this study elicited no significant degradation behavior as revealed by radiographs and micro-CT images. The interfaces of the Ti6Al4V alloy and PLA had good connection with the bone tissue, as well as with the connective tissue. However, Mg_60_Zn_35_Ca_5_ BMGC had a slightly rough appearance. Osteoblasts could be clearly visualized in the Mg_60_Zn_35_Ca_5_ BMGC group around the rod, a result which was compatible with ARS and von Kossa staining (Figure 2). At 24 weeks, the bone tissue of the Mg_60_Zn_35_Ca_5_ BMGC rod implants had much smoother interfacial morphologies and a much denser bone matrix than those achieved with the Ti6Al4V alloy and PLA rod implants. Overall, Mg_60_Zn_35_Ca_5_ BMGC demonstrated significantly greater osteo-promoting effects than those of the Ti6Al4V alloy and PLA groups in terms of ECM depositions and new bone formation, both radiographically and histologically.

However, it should be noted that there were some limitations in this study. First, after considering the economic and ethical issues of animal studies and the minimum number required to obtain valid results, three animals were included in each subgroup. This sample size of animals in each subgroup is likely insufficient. Second, since PLA implants are not radio-opaque, radiographic follow up was unable to provide much useful information. The position of the implants could only be validated after the animals were sacrificed.

## 4. Materials and Methods

### 4.1. Sample Preparation

Mg_60_Zn_35_Ca_5_ BMGCs with a 40% volume fraction of Ti particles 75–105 μm in diameter were prepared by induction melting under an argon atmosphere. First, high-purity Mg, Zn, and Ca (>99.9%), along with pure Ti particles, were melted together by induction melting under an argon atmosphere. During melting, the melt was churned mechanically to ensure that the final ingot contained a homogeneous mixture of Ti and other particles. Ingots of Mg_60_Zn_35_Ca_5_ BMGC were re-melted in a quartz tube and injected into a water-cooled Cu mold under an argon atmosphere to form Mg_60_Zn_35_Ca_5_ BMGC rods with a length of 10 mm and diameter of 2 mm. All surfaces were polished with #1200 sandpaper to ensure surface flatness and roughness. The chemical composition of the samples was verified by energy-dispersive spectroscopy (Inspect F50; FEI, Hillsboro, OR, USA) to confirm whether their compositions were the same as originally designed. Commercial medical-grade Ti6AL4V alloy and PLA were provided by President Co., Ltd. (Taipei, Taiwan) and BioTech One Co., Ltd. (Taipei, Taiwan). The samples were cut into rods with 10 mm length and 2 mm diameter, and polished at both ends to ensure surface flatness. Our previous studies have reported the microstructure, thermal properties, mechanical properties, and degradation behavior of Mg_60_Zn_35_Ca_5_ BMGCs with 40 vol% Ti particles (with particle size of 75–105 μm) [4,5]. Mg_60_Zn_35_Ca_5_ BMGCs with 40 vol% Ti particles possess the amorphous matrix and exhibit a compressive strength of around 800 MPa, and the calculated degradation rate around 0.26 mm/year.

### 4.2. In Vitro Test

#### 4.2.1. MTT Assay and Live/Dead Assay

Rabbit primary osteoblasts harvested from the pelvic bone were used to evaluate the biocompatibility of Mg_60_Zn_35_Ca_5_ BMGC, Ti6Al4V alloy, and PLA. The rabbit primary osteoblasts were cultured in high-glucose Dulbecco’s Modified Eagle Medium (DMEM; Gibco^®^, Carlsbad, CA, USA) supplemented with 10% fetal bovine serum (Gibco^®^, Carlsbad, CA, USA). Biocompatibility tests were then performed via the indirect contact method. In brief, all samples were immersed in DMEM solution, and the precipitate medium was collected at different time points (days 1, 3, 7, 14, and 30). A cell suspension (100 μL, 5000 cells/well) was dispensed in a 96-well culture plate that was pre-incubated for 24 h in an incubator at 37 °C and under a 5% CO_2_ atmosphere. After 24 h, the cells were attached to the culture plate, and 10 μL of the precipitate medium was dispensed into 96-well culture plates. Next, these were cultured in an incubator at 37 °C in a 5% CO_2_ atmosphere for another 24 h. A 10 μL MTT (3-(4,5-dimethylthiazol-2-yl)-2,5-diphenyltetrazolium bromide) solution (Invitrogen, Carlsbad, CA, USA) was then added carefully to each well, and the plates were incubated for 3 h. Dimethylsulfoxide (100 μL) was added, and the optical density was measured at 560 nm with an enzyme-linked immune-sorbent assay reader (Multiskan FC; Thermo, Waltham, MA, USA).

The live/dead cell viability was carried out by the double-staining method. The cells grown in a 24-well plate were then treated with Mg_60_Zn_35_Ca_5_ BMGC, Ti6Al4V alloy, or PLA precipitate medium at different times (days 1, 3, 7, 14, and 30). After 24 h, the cells were then washed with phosphate-buffered saline (PBS), added to a LIVE/DEAD assay kit (Thermo Fisher Scientific, Waltham, MA, USA), and incubated for 30 min. Images of cells were captured by a fluorescence microscope (IX81, Olympus, Tokyo, Japan).

#### 4.2.2. ALP Staining

The alkaline phosphatase activity (ALP) of rabbit primary osteoblasts was stimulated at certain times (days 3, 7, and 14) for the samples containing Mg_60_Zn_35_Ca_5_ BMGC, Ti6Al4V alloy, or PLA precipitate medium, and were tested by the staining method. First, 500 μL of cell suspension (1 × 10^4^ cells/well) was dispensed into 24-well culture plates and pre-incubated for 24 h in an incubator at 37 °C and under a 5% CO_2_ atmosphere. Second, 10 μL of the precipitate medium was added to the 24-well culture plates, and they were cultured in an incubator for 9 days, with the culture and precipitate media replaced after every three days. After 9 days of incubation, the medium was removed and fixed with 4% formaldehyde for 15 min.

The fixative was removed, and the samples were rinsed three times with DI water, and then 5-bromo-4-chloro-3-indolyl phosphate (BCIP)/nitro-blue tetrazolium (NBT) substrate (Sigma-Aldrich, Saint Louis, MO, USA) was added to each well. After five min of incubation, DI water was added to stop the reaction, and observation and imaging was carried out using an optical microscope (Primovert; Zeiss, Oberkochen, Germany). The quantitative results were analyzed and compared using automated Image J software.

#### 4.2.3. Alizarin Red S staining and Von Kossa Staining

Rabbit primary osteoblasts were used to evaluate the extracellular calcium deposited by the ions released from Mg_60_Zn_35_Ca_5_ BMGC, the Ti6Al4V alloy, and PLA. The precipitate medium contained the ions of the three materials collected at different times (at days 1, 3, 7, 14, and 30). The cell suspension (500 μL, 1 × 10^4^ cells/well) was dispensed into 24-well culture plates and pre-incubated for 24 h in an incubator at 37 °C and under a 5% CO_2_ atmosphere. Next, 10 μL of the precipitate medium was added to each of the 24-well culture plates, and they were cultured in an incubator for 72 h. The culture medium was then removed from each well, and the cells were gently washed with PBS (Gibco^®^) before they were treated with a fixative (4% formaldehyde) for 15 min at room temperature. The fixative was then removed, and the cells were washed three times with DI water.

For Alizarin red S (ARS) staining, 1 ml of 40 mM ARS reagent (Sigma-Aldrich, Saint Louis, MO, USA) was added to each well, which was then incubated at room temperature for 30 min. For von Kossa stain, 250 μL of 4% silver nitrate solution (Sigma-Aldrich, Saint Louis, MO, USA), was added to each well, and the cell container was placed in bright light until the calcium deposits turned black or dark brown. The cells were then rinsed three times with distilled water by gently shaking. The unreacted silver nitrate solution was rinsed using 250 μL of 5% sodium thiosulfate solution (Sigma-Aldrich, Saint Louis, MO, USA) and kept for five min at room temperature. After the staining process, the samples were observed and imaged by an optical microscope (Primovert; Zeiss, Oberkochen, Germany).

#### 4.2.4. Migration Test

The in vitro migration capacity of rabbit primary osteoblasts was assessed by transmembrane assay using 8 µm pore size inserts. The migration capacity of the rabbit primary osteoblasts was stimulated several times (on days 3, 7, and 14) for the Mg_60_Zn_35_Ca_5_ BMGC, Ti6Al4V alloy, and PLA precipitate media. First, cells were suspended in DMEM (Gibco^®^, Carlsbad, CA, USA) with a density of 2.5 × 10^4^ cells/mL per well and seeded with 200 µL in the inserts (one side of the membrane). Second, 750 µL of the precipitate medium was added to the opposite side (lower chamber) at varying times. After incubation at 37 °C for 12 h, the medium was removed from the inserts and the cells were gently washed twice in PBS. The cells were then treated with a fixative (4% formaldehyde) for two min at room temperature. Next, the fixative was removed, and the cells were washed twice with PBS before undergoing permeabilization with 100% methanol. After this, the cells were stained with crystal violet for 20 min at room temperature. The non-migrating cells were scraped off, and the migrated cells were captured and counted using an optical microscope (Primovert; Zeiss, Oberkochen, Germany) for comparison using automated Image J software.

#### 4.2.5. Cell Morphology Observation

The cell morphology of MG63 cell line were observation by scanning electron microscope (SEM) (SU3500; Hitachi, Tokyo, Japan). Mg_60_Zn_35_Ca_5_ BMGC rods with a diameter of 2 mm were polished to two parallel surface by 1200 Grit sandpaper. After sterilization, Mg_60_Zn_35_Ca_5_ BMGC samples were placed in to 24-well plate, the cell suspension (300 μL, 3000 cells/well) was dispensed into 24-well culture plate and incubated for four h to ensure cells were attached to Mg_60_Zn_35_Ca_5_ BMGC samples. After fixation and dehydration process, the cells were observed and captured by SEM.

### 4.3. In Vivo Test

#### 4.3.1. Experimental Design

All experimental protocols were in compliance with Animal Protection Act. Animal procedures were reviewed and approved by the Institutional Animal Care and Use Committee (IACUC) of Taipei Medical University (approval no. LAC-2017-0233; 05.02.2018). Eighteen male New Zealand white rabbits with a mean body weight of 3.5 ± 0.5 kg and an age of six months were randomly divided into three groups: Mg_60_Zn_35_Ca_5_ BMGC, Ti6Al4V alloy, and PLA. Each group was subdivided into two subgroups with implantation periods of 12 and 24 weeks. There were three animals in each subgroup.

#### 4.3.2. Surgical Method

Animal surgery was performed under general anesthesia induced by the intramuscular injection of tiletamine and zolazepam (15 mg/kg; Virbac, Carros, France), as well as xylazine hydrochloride (12 mg/kg; Bayer AG, Leverkusen, Germany). In both knee joints, a lateral parapatellar arthrotomy was performed on the femur condyles. A tunnel was drilled perpendicular to the long axis of the femur by an electrical bone drill with a diameter of 2.4 mm and length of 10 mm. The rods composed of Mg_60_Zn_35_Ca_5_ BMGC, Ti6Al4V alloy, or PLA were then inserted into the tunnel. The subcutaneous and skin layers were then sutured using 3-0 vicryl and nylon sutures (Ethilon, Norderstedt, Germany). After surgery, the rabbits were allowed unfettered movement and placed in independent cages. Antibiotic (enrofloxacin; 12 mg/kg; Bayer AG, Leverkusen, Germany) and analgesic (kentoprofen; 12 mg/kg; Nang-Kuang Pharmaceutical Co., Ltd., Taipei, Taiwan) medications were given before surgery and were continued for three days.

#### 4.3.3. Radiological Observation

Radiographs (anterior–posterior view) were taken every six weeks after surgery to examine the implant position and the possible generation of hydrogen gas cavities.

#### 4.3.4. Micro-CT Scan and 3D Image Reconstruction

The femoral bone of the rabbit was harvested after animal sacrifice and scanned by micro-computed tomography (µ-CT; Skyscan 1176; Bruker, Billerica, MA, USA). µ-CT scan of the samples was done at a voltage of 90 kV, at 8000 × 8000 pixels per slice, and at a resolution of 9 µm. After scanning, the micro-CT images were reconstructed with reconstruction software (NRecon Reconstruction; Bruker). Moreover, the bone density of the different implanted materials that were in use for different time periods (12 weeks and 24 weeks) was calculated using the reconstruction data and compared with a standard sample.

#### 4.3.5. Hematology Analysis

The animals were exsanguinated before they were sacrificed. The blood was centrifuged, and the serum was collected and frozen at −80 °C in a refrigerator before it was sent for biochemical blood analysis with a chemistry analyzer (VetTest; Idexx, Westbrook, ME, USA). The measurement items from the blood biochemical tests included alkaline phosphatase (ALKP), alanine aminotransferase (ALT), aspartate aminotransferase (AST), blood urea nitrogen (BUN), creatinine (CREA), l-lactate dehydrogenase (LDH), magnesium (Mg), and total bilirubin (TBIL).

#### 4.3.6. Histological Observation

The bone samples collected from the animals were formalin fixed and decalcified with a decalcification solution (10% formic acid and 10% HCl with distilled water). The decalcification solution was replaced every day until complete decalcification had occurred. Next, the samples were rinsed in distilled water and dehydrated using 70%, 80%, 95%, and 99.9% alcohol, for two h each. The samples were cleaned with xylene twice, immersed in paraffin for two h, and embedded in a paraffin block. Finally, the samples were sectioned and stained with hematoxylin and eosin (H&E) to visualize the bone morphology of the osteoblasts at the implanted site.

### 4.4. Statistical Analysis

All results are presented as mean ± standard deviation. All analyses were performed using SPSS20. The data for each group were analyzed using one-way analysis of variance followed by post hoc Scheffe tests. The differences between materials and amounts of time were analyzed by independent-sample t testing, and the statistical significance was set at a *p* value of <0.05.

## 5. Conclusions

In this study, Mg_60_Zn_35_Ca_5_ BMGC was systematically investigated by in vitro and in vivo tests and compared with Ti6Al4V alloy and PLA. The following conclusions may be drawn from our findings:

- At low concentrations of extraction medium treatments, the cell survival rates of osteoblasts on Mg_60_Zn_35_Ca_5_ BMGC, Ti6Al4V alloy, and PLA can be higher than 80%. According to ISO-10993-5 [10], all samples could be classified as having first level cytotoxicity (slightly toxic).
- Mg_60_Zn_35_Ca_5_ BMGC demonstrated excellent in vivo biocompatibility, and the osteogenic and osteoconductive potentials of these implants were superior to the conventional Ti6Al4V alloy and PLA.
- With an improved biodegradation rate, excellent biocompatibility, and most importantly, osteogenic ability, Mg_60_Zn_35_Ca_5_ BMGC has great potential for future surgical implant development and application.
